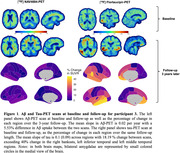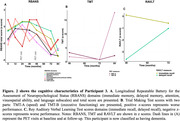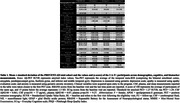# A‐T+ PET participants in preclinical AD: Clinical progression and concordance with fluid markers

**DOI:** 10.1002/alz.094046

**Published:** 2025-01-09

**Authors:** Yara Yakoub, Frédéric St‐Onge, Alfonso Fajardo, Bery Mohammediyan, Christine Dery, Elisabeth Sylvain, Jennifer Tremblay‐Mercier, Jordana Remz, Julie Gonneaud, Jacob W. Vogel, Jean‐Paul Soucy, Alexa Pichet Binette, Sylvia Villeneuve

**Affiliations:** ^1^ Douglas Mental Health University Institute, Centre for Studies on the Prevention of Alzheimer's Disease (StoP‐AD), Montréal, QC Canada; ^2^ Clinical Memory Research Unit, Lund University, Lund Sweden; ^3^ Montreal Neurological Institute, McGill University, Montréal, QC Canada

## Abstract

**Background:**

Amyloid‐negative tau‐positive PET (A‐T+) participants have been reported in several studies. We assessed the prevalence and characteristics of A‐T+ participants in a cohort of cognitively unimpaired individuals with a first‐degree family history of Alzheimer’s disease (AD) dementia.

**Method:**

We studied 252 participants from the longitudinal PREVENT‐AD cohort (mean cognitive follow‐up = 3.42 years, SD = 2.86) who received an amyloid (18F‐NAV4694) and a tau (18F‐Flortaucipir) PET‐scan. The amyloid‐positivity threshold was 18 centiloids (SUVR=1.27) and tau positive threshold was set as 2SD above the mean of A‐ participants in a temporal meta‐ROI (SUVR=1.29). Follow‐up PET scans were available for 109 participants. We characterized A‐T+ participants on clinical (age, sex, APOE4 status, education, depression, apathy, anxiety, sleep), cognitive (RBANS, MMSE, ECoG) and fluid (CSF, plasma) measurements.

**Result:**

In the PREVENT‐AD cohort, only 3 (1.2 %) of all studied participants were classified as A‐T+ (see Table 1 for participants characteristics). Participants 1 and 2 were positive based on plasma Aß42/40 and they had relatively low levels of tau binding that could represent early pathology. Participant 3 was also positive based on CSF and plasma, her Aß‐PET scan was classified as positive on visual read and became quantitatively positive at follow‐up. Participant 3 had extensive unilateral tau binding at baseline that became bilateral at follow‐up (Figure 1). Figure 2 details the longitudinal cognitive trajectory of Participant 3. During the cognitive follow‐up, participants 1 developed mild cognitive impairment and participant 3 developed dementia.

**Conclusion:**

A‐T+ individuals are rare in the PREVENT‐AD cohort and the 3 A‐T+ were classified as Aß positive based on fluid biomarkers. One of the 3 A‐T+ individuals showed a very fast clinical progression and a tau uptake pattern atypical of AD.